# Economic Evaluation of Pneumococcal Vaccination in Egypt: Cost-Effectiveness, Budget Impact, and Domestic Manufacturing Potential

**DOI:** 10.3390/vaccines14040318

**Published:** 2026-04-01

**Authors:** Chrissy Bishop, Arnold Hagens, Federico Rodriguez-Cairoli, Konstantina Politopoulou, Zicheng Wang, Motuma Abeshu, Sowmya Kadandale, Ibironke Oyatoye, Saadia Farrukh

**Affiliations:** 1 Triangulate Health Ltd., Doncaster DN11 9QU, UK; 2 Department of Health Sciences, University Medical Center Groningen, University of Groningen (RUG), 9713 AV Groningen, The Netherlands; 3 UNICEF MENA Regional Office, Abdulqader Al-Abed Street, Building No. 15 Tla’a Al-Ali, Amman 11821, Jordan; 4 UNICEF Headquarters, 3 UN Plaza, Room 816, New York, NY 10017, USA

**Keywords:** pneumococcal vaccination, Egypt, cost-effectiveness, budget impact, dynamic model

## Abstract

**Background/Objectives:** Streptococcus pneumoniae remains a major cause of morbidity and mortality in Egypt, yet pneumococcal conjugate vaccines (PCVs) are not included in the national immunization program. Recent commitments to domestic vaccine manufacturing and temporary Gavi support create a timely decision context for policymakers to assess whether PCV introduction is cost-effective, affordable, and sustainable within Egypt’s health financing constraints. This study evaluates the cost-effectiveness, budget impact, and return on investment (ROI) of PCV introduction in Egypt. **Methods:** A deterministic, age-structured dynamic transmission model was developed to estimate the health and economic outcomes of PCV introduction over a 20-year horizon from a healthcare payer perspective. The analysis was conducted in line with the Consolidated Health Economic Evaluation Reporting Standards 2022 (CHEERS 2022) guidelines. The model captures direct and indirect effects across all age groups and includes pneumonia, meningitis, non-pneumonia non-meningitis invasive disease, and acute otitis media. Scenarios assessed immediate versus delayed introduction, alternative PCV10-to-PCV13 pathways, and domestic manufacturing price assumptions. Outcomes included deaths averted, incremental cost-effectiveness ratios (ICERs) relative to GDP per capita, budget impact, and ROI using the value of statistical life. **Results:** Immediate PCV13 introduction was projected to avert 139,451 deaths across all age groups over 20 years, with an ICER of 523.31 USD per DALY averted equal to 0.16 × GDP per capita. The total budget impact was USD 124.9 million per year without Gavi support and USD 120.9 million with support, yielding an ROI of 23.1. Delaying the introduction substantially reduced health gains and economic returns. Pathways involving initial PCV10 introduction followed by transition to PCV13 achieved similar health outcomes with a lower budget impact and higher ROI. **Conclusions:** PCV introduction in Egypt represents a high-value investment. Immediate introduction maximizes health and economic benefits, while delayed introduction entails substantial opportunity costs. Alternative PCV10-to-PCV13 pathways offer a more affordable route with a similar long-term impact.

## 1. Introduction

*Streptococcus pneumoniae* (S. Pn) is a Gram-positive, encapsulated bacterium and a major cause of an array of diseases such as pneumonia, acute otitis media, and meningitis. Transmission generally occurs through direct person-to-person contact via respiratory droplets through coughing and sneezing. In addition, people can be carriers without becoming unwell and possibly spread the bacteria to others. It is estimated that worldwide, approximately one million children die every year due to S. Pn. [1]. In Egypt, the Global Burden of Disease (GBD) estimated 6500 deaths from S. Pn in 2021. Many of these deaths can be prevented through vaccination. The 13-valent pneumococcal conjugate vaccine (PCV13) protects against 13 types of S. Pn. bacteria and has an effectiveness of around 45% for all-cause pneumonia [2]. PCV13 therefore can reduce morbidity and mortality, and as a consequence, related economic effects such as healthcare costs.

Vaccinating against S. Pn. leads to a range of positive impacts; however, it also entails costs. These include vaccine procurement, program coordination, and the resources required for vaccine introduction into the routine immunization program. It is therefore essential to understand the expected health and economic effects before introducing the vaccine. Experiences in other countries show that PCV introduction consistently leads to substantial health gains, with evaluations reporting ~860 deaths averted per year in childhood-focused models [3], and ~2490 deaths per year in a 100-year modelling horizon [4]. Global evidence in low- and middle-income countries (LMICs) further suggests 1000–1800 preventable deaths annually for countries of Egypt’s size (116 million in 2024) [5] and demonstrates that PCV programs are highly cost-effective, with ICERs typically 0.2–0.6× GDP per capita [6].

Vaccinations against S. Pn are not currently part of Egypt’s national immunization program. The decision to introduce them was initially encouraged by a national cost-effectiveness analysis conducted by Sibak et al. in 2013 [3], which found PCV13 to be cost-effective (USD 3916 per DALY averted). The 2013 national cost-effectiveness analysis is now outdated, having used earlier disease burden estimates and willingness-to-pay thresholds and omitted indirect (herd immunity) effects. Since then, the decision context has also shifted. In December 2021, the Egyptian President issued a directive to establish the Vaccines and Biotechnology City in Egypt to strengthen domestic vaccine production [7]. PCV13 was identified as one of the priorities for domestic production. In parallel, Egypt can leverage external financing and technical support from the Gavi Alliance middle-income country scheme and UNICEF procurement mechanisms, creating near-term options for vaccine introduction while domestic capacity is developed [8]. Against this backdrop, updated economic evidence is needed to inform a time-sensitive choice among plausible introduction and supply pathways. Decision-makers must weigh (i) introduction via continued procurement through UNICEF (with temporary Gavi support where applicable), (ii) a phased transition from imported to domestically produced PCVs, and (iii) alternative product pathways (PCV10 initially, with a later switch to PCV13), recognizing that establishing local production typically requires 5–10 years [9,10]. This study therefore updates and extends the earlier national analysis to reflect current epidemiology, costs, and Egyptian industrial development priorities. Further, the timing, affordability, and economic value of PCV introduction are compared under these alternative procurement and domestic manufacturing scenarios.

Therefore, the main research question to be answered is as follows: “What is the cost-effectiveness and budget impact of introducing PCV in Egypt compared with no PCV under certain scenarios?” To be able to answer this, the cost-effectiveness, return on investment, and budget impact of introducing PCV for children under five versus no vaccination are assessed using a dynamic transition model. Dynamic transmission models are essential for PCV economic evaluation because they capture herd protection effects and serotype replacement [11], phenomena that static cohort models cannot represent. This ensures that the full epidemiological impact on cases, deaths, DALYs, and costs across all age groups is accurately reflected in cost-effectiveness estimates. Cost-effectiveness results summarize the cost per health gain, while ROI results capture the broader economic returns associated with reduced disease burden. Budget impact results summarize the expected financing implications for government and other payers, informing affordability and sustainability under constrained health budgets.

## 2. Materials and Methods

### 2.1. Overview

This analysis was structured and reported in accordance with the CHEERS 2022 guidelines for health economic evaluations [12]. The cost-effectiveness of PCV introduction in Egypt from a health system perspective was studied. A deterministic age-structured, dynamic compartmental model was developed to evaluate the cost-effectiveness of introducing PCV in Egypt over a 20-year time horizon. The model simulates pneumococcal transmission and disease progression across the entire population and was selected to capture both direct effects in children and indirect (herd immunity) effects in individuals aged >5 years, as well as the way these effects evolve following vaccine introduction. The core transmission component follows a standard SIRD framework (Susceptible–Infectious–Recovered–Death) [13], formulated as a system of ordinary differential equations (ODEs) and implemented in continuous time, in which the number of new infections depends on the interaction between susceptible and infectious individuals. The model was adapted to reflect the epidemiology of *Streptococcus pneumoniae* and the mechanism of vaccine-derived protection. The population is divided into three immunity groups: susceptible unvaccinated, susceptible vaccinated, and recovered-immune. Individuals in each group progress through the same health state: susceptible, infectious, disease, and either recovery or death. Natural immunity following infection provides partial protection, while vaccine-induced immunity confers higher protection [14,15]. Both forms of immunity wane over time, allowing individuals to return to the susceptible pool.

**Figure 1:**
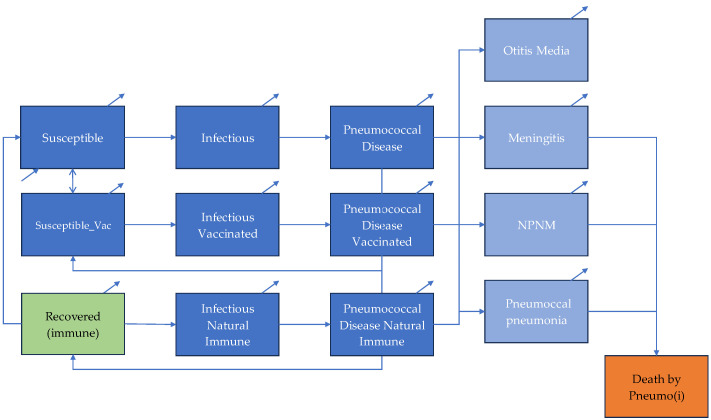
Schematic overview of pneumococcal model (small arrows represent births and background mortality). Dark blue = all transmission/progression compartments across the entire population, light blue = clinical disease outcomes, green = recovered (immune), orange = death.

The disease state is disaggregated into four pneumococcal syndromes: acute otitis media (AOM), pneumococcal pneumonia, meningitis, and non-pneumonia non-meningitis (NPNM) invasive disease, including sepsis. Age-specific incidence, case fatality, and severity parameters determine transitions between infectious and disease states. Long-term sequelae were not specifically included to keep model outcomes conservative. Demographic processes, including births and background mortality, are incorporated to reflect population turnover and maintain epidemiological dynamics over the 20-year time frame. A schematic illustration of the transmission pathways, immunity transitions, and disease compartments is presented in Figure 1.

### 2.2. Dynamic Transmission Model

The dynamic transmission component operates across three age groups (0–4 years, 5–64 years, and ≥65 years). Age-specific transmission patterns were modelled using a contact matrix derived from Prem et al. [16], adjusted to reflect the demographic structure of Egypt. The force of infection in each age group depends on the number of susceptible individuals and the number of infectious carriers (vaccinated, unvaccinated, and recovered-immune), weighted by age-specific contact rates.

The transmission dynamics are governed by a system of differential equations that simulate transitions between susceptibility, infection, and recovery for vaccinated and unvaccinated individuals (Appendix A provides transmission and progression equations).

The disease transmission model was developed and implemented in RStudio (version 2024.4.2.764, Posit PBC).

### 2.3. Model Assumptions and Analytical Framework

Table 1 summarizes the core modelling assumptions underpinning the economic evaluation, including the model type, analytic perspective, time horizon, discount rate, and comparator. Economic parameters were selected to reflect Egypt’s health financing context and follow international guidance. Both PCV10 and PCV13 were included because they have substantially different price points but broadly similar effectiveness profiles, as noted by WHO SAGE [17]. Evaluating both products enabled assessment of how vaccine pricing influences cost-effectiveness. The eligible population—children from birth to one year—follows WHO-recommended schedules for pneumococcal vaccination [18]. A 3-dose vaccine schedule was analyzed without a booster (3 + 0), reflecting a pragmatic schedule for early implementation and following guidance from UNICEF. Vaccine coverage assumptions were based on historical DTP3 coverage in Egypt and validated with UNICEF Egypt data, ensuring realistic projections of PCV uptake.

A healthcare payer perspective was adopted because pneumococcal vaccine procurement and delivery are financed primarily through government budgets in Egypt, consistent with prior PCV evaluations in LMIC settings [3,4]. Direct medical costs were therefore used to reflect decision-relevant budgetary impacts. A 20-year time horizon was used to capture the long-term health and economic consequences of vaccine introduction, including the gradual accumulation of both direct and indirect effects. Future costs and outcomes were discounted at an annual rate of 3.5%, in line with local economic evaluation guidelines [19]. Egypt-specific willingness-to-pay thresholds were applied to provide contextually appropriate interpretation of cost-effectiveness [20].

Table 2 summarizes the epidemiological, clinical, and vaccine-related parameters incorporated into the dynamic model. Age-specific incidence rates and case-fatality ratios for pneumococcal pneumonia, meningitis, AOM, and NPNM were drawn from Egyptian economic evaluations [3,4] and adjusted to remain consistent with Global Burden of Disease estimates [1], providing locally relevant disease burden inputs.

Vaccine effectiveness estimates were based on Sibak et al. [3] and then refined using PCV13 serotype coverage data from Badawy et al. [21]. To reflect expected post-introduction epidemiology, vaccine effectiveness values for invasive disease were further adjusted for serotype replacement using evidence from Weinberger et al. [22]. The model also applies a 20% reduction in vaccine-targeted serotype coverage for invasive pneumococcal diseases (IPDs) over the analysis period to account for replacement, ensuring realistic long-term impact estimates.

As natural immunity after infection is serotype-specific and short-lived, serotype prevalence ranges widely and multi-serotype carriage is common. Based on these considerations [15,22,23], natural immunity was conservatively set at 33% of vaccine-induced protection, high enough to account for some cross-protection but low enough to avoid underestimating the incremental value of vaccination. An assumed reproduction number (R_0_) of 1.25 was used to reflect pneumococcal transmission dynamics in high-burden settings. This value is consistent with estimates reported in the literature, where pre-vaccine R_0_ for *Streptococcus pneumoniae* typically ranges from 1.2 to 1.4 depending on serotype distribution, population density, and age-mixing patterns [24]. The derived R_0_ in combination with the contact matrix was calibrated with the real reported incidence rates per included disease. Although we used an average R_0_, the effects per age group differed through the contact matrix and calibration.

These inputs ensure that transmission dynamics, vaccine effects, and disease outcomes reflect both local epidemiology and established biological mechanisms.

**Table 1:** Summary of model structure, inputs, and economic evaluation assumptions.

Category	Variable/ Parameter	Value/Description	Source
Model characteristics	Model type	Dynamic transmission model	Decision made by the authors
	Time horizon	20 years	
	Perspective	Healthcare payer and societal	
	Discount rate	3.5% per year for costs and health benefits	[19]
Diseases in model	Pneumococcal syndromes	Acute otitis media, meningitis, NPNM disease, and pneumonia	[3]
Disutility values	Disability weight for each condition	AOM: 0.05 Meningitis, NPNM disease, and pneumonia: 0.13	[1]
Healthcare resource use	Direct medical costs	See cost table for disease-specific costs	Decision made by the authors
Intervention characteristics	Target population	Children from birth to 1 year (WHO PCV schedules)	[18]
	Vaccine products modelled	PCV10 and PCV13	Decision made by the authors
	Vaccine coverage	Determined by scenarios (immediate introduction, 5-year delay, etc.)	
	Total number of doses	3 doses (WHO recommendation)	[18]
	Program cost per fully vaccinated child	Varies by local vs. imported production; see cost table	[25,26,27,28]
Main outcomes	Health metrics	Deaths and disability-adjusted life years (DALYs) avoided	Decision made by the authors
Economic evaluation	Metrics	Incremental cost-effectiveness ratio (ICER), budget impact, total vaccination costs, return on investment	
Willingness-to-pay thresholds	WTP thresholds	0.20, 0.39, 0.49 × GDP per capita	[20]
Sensitivity analyses	One-way analysis	Key parameters varied by ±20%	Decision made by the authors
	Scenario analyses	Vaccine introduction timing and price scenarios

**Table 2:** Input parameters by age group.

Parameter	0–4	5–64	65+	Reference
Transmission parameters				
Basic reproduction number	1.25	1.25	1.25	[24]
Estimated VE on infection	72%	72%	72%	Assumed to be the same as serotype coverage [21]
Disease—Otitis media				
CFR	0	0	0	[3]
Average incidence/100,000 persons	9970	839	0.00	[4]
VE	57%	Not apply	Not apply	[29]
Effectiveness natural immunity	0.19	0.19	0.19	[15,22,23] *
Disease—Meningitis				
CFR	0.08	0.02	0.05	[3]
Average incidence/100,000 persons	4.34	0.27	0.05	[4]
VE	48%	Not apply	Not apply	[3,21,22] **
Effectiveness natural immunity	0.16	0.16	0.16	[15,22,23] *
Disease—NPNM				
CFR	0.05	0.05	0.05	[3]
Average incidence/100,000 persons	15.66	2.67	1.92	[4]
VE	0.48%	Not apply	Not apply	[3,21,22] **
Effectiveness natural immunity	0.16	0.16	0.16	[15,22,23] *
Disease—Pneumococcal pneumonia				
CFR	0.03	0.01	0.04	[4]
Average incidence/100,000 person	857	267	1116	[4]
VE	0.59%	Not apply	Not apply	[4]
Effectiveness natural immunity	0.19	0.19	0.19	[15,22,23] **

Note: VE: Vaccine effectiveness. CFR: Case-fatality rate. * Assumed 33% of VE. ** Calculated by multiplying PCV13 vaccine effectiveness against pneumococcal pneumonia/meningitis/NPNM from Sibak et al. [3], with PCV13 serotype coverage from Badawy et al. [21], with serotype replacement on IPD for children < 5 from Weinberger et al. [22].

### 2.4. Cost and Effects

Budget impact and cost-effectiveness were assessed over the 20-year modelling horizon. Only direct healthcare costs were included, defined as the average cost per incident case of each pneumococcal syndrome (Table 3), alongside vaccine procurement and delivery costs modelled under several price scenarios (Appendix B). Procurement and delivery costs incorporated standard components used in global immunization costing, including delivery unit costs, freight and handling fees, injection supplies, surcharge structures, and human resource inputs, drawing on WHO and UNICEF Supply Division methodologies and country-level delivery cost databases [25,26,27,30], with additional verification from UNICEF SD.

(1) $$ICER=\frac{Costs\;scenario-Costs\;BAU\;scenario}{DALYs\;BAU-DALYs\;\;scenario}$$

Health effects were measured in disability-adjusted life years (DALYs), with each cycle calculated as years of life lost plus years lived with disability. Incremental cost-effectiveness ratios (ICERs) were computed by dividing incremental costs by the DALYs averted relative to the business-as-usual (BAU) scenario (Equation (A1)) To estimate the cost-effectiveness, the ICER/GDP per capita ratio was used. Vaccine procurement costs were set at USD 16.00 per dose for PCV13 and USD 3.45 for PCV10 [31], resulting in total program costs of USD 68.00 and USD 20.57 per fully vaccinated child, respectively.

Adverse events following immunization were not modelled in the analysis. This decision was based on the assumption that severe adverse events associated with both PCV10 and PCV13 vaccination are uncommon, in line with the decision made by existing economic evaluations [32,33].

**Table 3:** Cost inputs (in 2024 USD).

Parameter	Age 0–4	Age 5–64	Age ≥ 65	Reference
Cost per disease case—Otitis media	16.70	20.04	20.04	Estimated using PPP-adjusted and inflation-corrected average prices from Tunisia and Algeria [34]
Cost per disease case—Meningitis	815.04	978.05	978.05	As above
Cost per disease case—NPNM	326.08	391.30	391.30	As above
Cost per disease case—Pneumococcal pneumonia	42.62	51.25	51.25	As above

### 2.5. Calibration

The model was calibrated to reproduce a realistic BAU scenario prior to vaccine introduction. To do this, a 21-year burn-in period was used to stabilize the susceptible, recovered, infectious, and disease levels and align the simulated population with Egypt’s demographic structure in the first analysis year. Calibration targets included reported incidence rates for acute otitis media, meningitis, non-pneumonia/non-meningitis invasive disease, and pneumococcal pneumonia. Model parameters were iteratively adjusted until simulated age-specific incidence patterns matched observed BAU values.

Population dynamics were incorporated through a demographic transition matrix that moves individuals into older age groups or death over time, ensuring that age-specific transmission patterns evolve with the underlying population structure.

The model was implemented in R (version 4.2), with Excel used for supplementary tabulation and diagramming. Numerical integration of the differential equations was performed using the classical fourth-order Runge–Kutta (rk4) method, implemented via the deSolve package in R.

### 2.6. Scenario Analysis Reflecting Egyptian Context

To address the question of affordability and sustainable financing, reflecting the Egyptian context, the analysis explored various scenarios based on two key variables, the time of introduction (discussed here) and the cost of vaccination (Section 2.4). Firstly, the BAU scenario reflects the current situation in Egypt, where no S. Pn vaccination occurs. The intervention scenarios vary the time of vaccination introduction between immediate, 5-year delay, and 10-year delay. Regarding vaccine products, as PCV13 is the priority vaccine product for Egypt [7], it was included in all modelled scenarios. To assess the price implications of introducing a lower-cost alternative, we also modelled two scenarios where immediate introduction of UNICEF-procured PCV10 was introduced, followed by either a switch or a gradual transition to domestically produced PCV13. For all scenarios that include UNICEF procurement, Gavi MICs support is incorporated.

### 2.7. Domestic Manufacturing Cost Assumptions

In addition to the 5 scenarios, the analysis considered varied domestic manufacturing cost assumptions. The analysis used the UNICEF long-term arrangement (LTA) price as a benchmark and explored potential deviations due to local manufacturing. The analysis allowed domestic manufacturing prices to be either higher or lower than the UNICEF LTA price, reflecting insights from both experts and the literature. Some evidence suggests local production could raise costs [35], while expert opinion highlights potential cost reductions through economies of scale and lower logistics expenses [36]. The UNICEF LTA price was therefore used as a benchmark, and domestic manufacturing prices were estimated at a −10%, 0%, and +10% change. For the immediate initiation, two additional scenarios were defined. One with the change from PCV10 pricing to PCV13 pricing in year 5, and a second with a gradual change in three years of change from PCV10 to PCV13. Table 4 gives an overview of the scenarios.

### 2.8. Sensitivity Analysis

To test the robustness of the model and outcomes, a one-way deterministic sensitivity analysis was performed on the cost of vaccination, vaccination uptake, years of life lost at premature death, vaccination effectiveness on transmission, and effectiveness on transmission for natural immunity. In addition, the following parameters were included for every disease, including cost of treatment, disutility, duration, case-fatality rate, vaccine effectiveness, and incidence. The key parameters were varied by ±20% in order to determine the impact. Results are presented for each parameter increased by 20% and decreased by 20%, respectively. For parameters that applied to all three age groups, the values were varied simultaneously, generating a single sensitivity analysis outcome. The sensitivity was performed on the immediate vaccination scenario with 0% change on the vaccination cost.

## 3. Results

Results are first presented for the base-case analysis of immediate PCV13 introduction, followed by comparative scenario and sensitivity analyses. Outcomes are reported as deaths averted, and ICERs are expressed relative to GDP per capita, annual budget impact, and return on investment over a 20-year time horizon.

### 3.1. Base-Case Cost-Effectiveness, Budget Impact, and ROI

In the base-case scenario of immediate PCV13 introduction at the UNICEF LTA price, vaccination is projected to avert 38.5 million cases and 139,451 deaths over 20 years compared with business as usual (Table 5). The ICER/GDP ratio of 0.16 indicates that the intervention is highly cost-effective.

The base-case results in a total annual budget impact of USD 124.91 million without Gavi support and USD 120.94 million with Gavi support. The program delivers a return-on-investment of 23.12, reflecting substantial savings from reduced pneumococcal disease burden.

These findings form the benchmark for interpreting all subsequent scenario and sensitivity analyses.

Across all scenarios, earlier introduction of pneumococcal vaccination produced greater health impact than delayed implementation (Table 6). Relative to the base case, a 5-year delay reduces deaths avoided from 139,451 to 97,374 (42,077 fewer deaths; 30.2% reduction). A 10-year delay further reduces deaths avoided to 54,407 (85,044 fewer deaths; 61.0% reduction) over 20 years.

Variation in domestic manufacturing price assumptions leads to modest increases in ICER/GDP ratios; however, all scenarios remain highly cost-effective. Differences are driven primarily by changes in program cost, as health outcomes remain comparable across price levels (Table 6).

Alternative product pathways also perform favourably relative to the base case. Scenarios involving immediate PCV10 introduction followed by PCV13—via a direct switch in year 5 or a gradual transition—result in ICER/GDP ratios of 0.12 and 0.13, respectively, reflecting lower initial vaccination costs while achieving similar long-term health outcomes. Overall, the scenario results indicate that delaying PCV introduction substantially diminishes both health and economic benefits, while immediate introduction whether with PCV13 or a temporary PCV10 pathway provides the highest value.

### 3.2. Age-Specific Distribution of Healthcare Cost Savings

**Figure 2:**
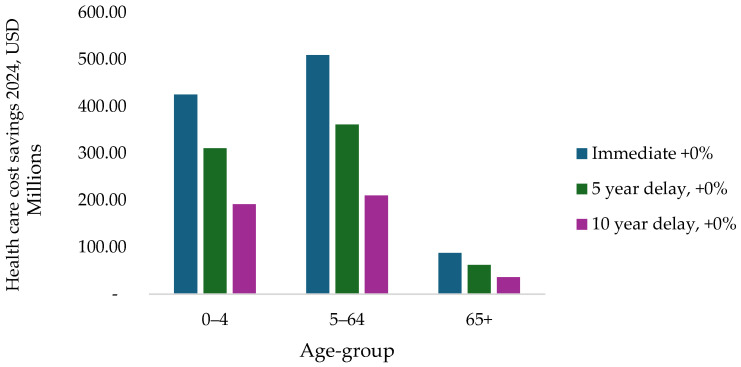
Impact of vaccination on the healthcare costs averted per age group (2024, USD).

Figure 2 presents the distribution of healthcare cost savings by age group across the immediate introduction, 5-year delay, and 10-year delay scenarios using the base PCV13 price. Immediate introduction produces the largest cost savings in all age groups. The greatest reductions occur in the 0–4 and 5–64 age groups, reflecting their higher burden of pneumococcal disease. Savings in the 65+ age group are smaller in absolute terms but remain positive across all scenarios. Overall, a substantial share of total healthcare cost savings occurs among individuals aged over five years.

### 3.3. Budget Impact

Table 7 presents the annual average budget impact of pneumococcal vaccination across all scenarios, with and without Gavi support, as well as the corresponding return on investment (ROI) over the 20-year horizon. Immediate introduction of PCV13 results in the highest budget expenditure but also the greatest long-term savings, reflected in the highest ROI among the PCV13 scenarios.

Delaying introduction reduces the overall budget impact; however, these scenarios are associated with lower ROI values compared with the base case. Alternative product pathways show favourable performance, with immediate PCV10 introduction followed by a switch to PCV13 yielding the highest ROI and the lowest budget impact within the immediate-introduction scenarios.

### 3.4. Sensitivity Analysis Results

One-way sensitivity analysis (Appendix C) shows that vaccine price and vaccination uptake are the two parameters with the greatest influence on cost-effectiveness and budget impact outcomes. A ±20% change in vaccine price results in annual average costs ranging from approximately USD 91 million to USD 159 million, compared with around USD 125 million in the base case. A ±20% change in vaccination uptake produces annual budget impacts between approximately USD 96 million and USD 131 million.

Other parameters, including disease-specific incidence, case-fatality rates, and disutility values, produced comparatively smaller changes in outcomes. Overall, the sensitivity analysis shows that model results are most responsive to changes in program cost and uptake levels.

As an internal validation step, we modelled a direct-effects-only scenario (excluding herd protection). This reduced total deaths averted to approximately 46,000 over 20 years (≈2300 per year), with corresponding ICERs of 0.27–0.38× GDP per capita and an ROI of USD 6–8 per dollar invested (Appendix C).

## 4. Discussion

This dynamic, age-structured cost-effectiveness analysis shows that introducing PCVs into Egypt’s routine immunization schedule is highly cost-effective and yields substantial health and economic benefits. Across all implementation scenarios, the incremental cost-effectiveness ratios (ICERs) were well below commonly used willingness-to-pay thresholds, and the ROI ranged from roughly US$16 to US$28 per dollar spent. Immediate introduction of PCV13 was projected to avert about 38.5 million cases and 139,451 deaths over twenty years, or nearly 6972 deaths per year, and to generate significant savings in healthcare expenditure through reductions in pneumonia, meningitis, non-pneumonia non-meningitis invasive disease, and acute otitis media (AOM).

Earlier Egyptian economic evaluations of PCVs have produced more conservative estimates of health gains and higher cost-effectiveness ratios. Sibak et al. [3] evaluated PCV13 using a static model that restricted benefits to children under five years and estimated roughly 800–2500 deaths averted per year, with ICERs between 0.26 and 1.2 times of Egypt’s gross domestic product (GDP) per capita [3]. Sevilla et al. employed a pseudo-dynamic Markov model over a 100-year horizon and reported about 2300 deaths averted per year with ICERs near one times GDP per capita [4]. A global pseudo-dynamic analysis across 112 LMICs by Chen et al. suggested that PCV13 introduction could avert approximately 697,000 deaths and 46 million disability-adjusted life years (DALYs) globally, with an incremental cost of 851 international dollars per DALY averted; scaled to Egypt, this implies 1000–1800 preventable under-five deaths per year [6]. Other LMIC analyses that account for indirect protection report cost-effectiveness ratios of about 0.20 × GDP per capita and above [11,37,38]. The direct-effects-only sensitivity analysis further supports the validity of the model. Under this scenario, the model projects ~46,000 deaths averted over 20 years (≈2300 annually), with ICERs of 0.27–0.38× GDP per capita more closely matching estimates from earlier Egypt-specific evaluations and global LMIC analyses of under-five mortality [3,4,6]. This concordance indicates that the higher mortality reductions observed in the full dynamic model arise from the inclusion of older age groups and indirect protection rather than from overly optimistic assumptions. Compared with these studies, our model predicts substantially greater health gains and more favourable cost-effectiveness.

Several methodological features help explain why this dynamic model produces larger estimated health and economic benefits than earlier Egyptian evaluations [3,4]. Unlike previous studies that focused primarily on children under five or used static herd-immunity-effect assumptions, our model applies the nationally adjusted disease burden across all age groups and explicitly simulates transmission, capturing substantial indirect protection in older children and adults. The 20-year horizon concentrates outcomes within a policy-relevant time frame, whereas longer horizons dilute annualized effects. Inclusion of acute otitis media and outpatient pneumonia, major contributors to morbidity and cost [39], also increases overall gains compared with models limited to invasive disease. We additionally assume more conservative natural immunity (one-third of vaccine-derived protection), which increases the incremental benefit of vaccination [15,22,23]. Finally, updated 2024 cost data and inclusion of the value of prevented premature deaths yield higher return-on-investment estimates than earlier analyses based mainly on direct medical costs.

### 4.1. Policy Implications and Optimal Introduction Strategy

The base case of immediate PCV13 introduction using UNICEF long-term agreement pricing produces the largest health gains and strongest economic returns. Delaying introduction by five or ten years reduces deaths averted by approximately 30% and 61%, respectively, and lowers ROI, even though delayed scenarios require lower annual budgets in absolute terms. Variation in domestic manufacturing prices only has a modest effect on ICERs; however, lower local prices improve ROI. Scenarios that begin with PCV10 procurement through UNICEF and then transition to locally manufactured PCV13 achieve health benefits comparable to immediate PCV13 introduction and yield the highest ROIs and the lowest annual budget requirements. These findings suggest that a dual-track approach, early adoption through international procurement, followed by a gradual shift to domestic production, can balance immediate health gains with long-term fiscal sustainability and industrial policy objectives.

PCV10 is only slightly less effective against currently prevalent serotypes but may offer lower long-term protection because of serotype replacement [40]. Its price, however, is roughly 80% lower than PCV13 [32], making it an attractive alternative when budget constraints preclude immediate PCV13 adoption. In such circumstances, implementing PCV10 first and transitioning to PCV13 once local manufacturing is established can still deliver strong health benefits and economic value.

Our pricing assumptions were intended to capture higher unit costs during the early phases of domestic production and lower costs once manufacturing reaches scale and enables regional export, operationalized as ±10% relative to the UNICEF long-term agreement price. This approach is supported by evidence showing that domestically manufactured vaccines are often more expensive per dose in their early years than vaccines procured through Gavi or UNICEF pooled mechanisms [41,42]. This pattern has been observed for PCVs and other vaccines, reflecting high upfront R&D and technology-transfer costs, a limited initial production scale, and restricted early market access [41,43]. In South Africa, local fill-and-finish production of PCV13 was reported to be more expensive than an imported alternative, leading to a subsequent switch [43]. While realized prices will depend on country-specific production arrangements and scale, these scenarios reflect a plausible, evidence-informed range rather than precise price forecasts.

The annual average budget impact of PCV as a proportion of the latest available annual healthcare expenditure for Egypt (2022) is 0.46–0.47% [5]. For comparison, the latest vaccine expenditure data (2022) suggests that Morocco and Tunisia spend 0.07% and 0.24% of annual healthcare expenditure, respectively, on all vaccines combined [44]. Compared to international benchmarks, the budget impact for all PCV scenarios in Egypt falls within the spending range for vaccine investments, which typically account for 0.4–1.4% of total health expenditure [45,46,47,48].

### 4.2. Strengths, Limitations, and Generalizability

Several notable strengths characterize this work. The age-stratified dynamic compartmental model captures heterogeneous mixing patterns and age-specific susceptibility profiles, while simultaneously modelling multiple infectious diseases within a unified framework. The exploration of multiple scenarios allows for a comprehensive assessment of uncertainty and potential future trajectories. Incorporating demographic ageing ensures that the model reflects evolving population structures over time. Calibration against real-world incidence data enhances empirical validity, and the integration of a social contact matrix provides a biologically grounded foundation for estimating age-assortative transmission.

This analysis has some limitations. Estimates of incidence and case fatality were derived from older published Egyptian and regional sources rather than comprehensive up-to-date Egyptian surveillance data and may therefore reflect measurement uncertainty. Assumptions regarding natural immunity, serotype replacement, and the duration of vaccine-derived protection may also differ from real-world dynamics. Indirect societal costs were not included, suggesting that the cost-effectiveness of PCV introduction may be conservative. Although the dynamic model captures herd immunity effects within Egypt, generalizability to other settings will depend on local epidemiology, coverage levels, and cost structures. The model also did not incorporate a one-time catch-up campaign recommended by SAGE [18], though its exclusion is unlikely to materially affect 20-year results. A further limitation is that a probabilistic sensitivity analysis was not performed, owing to the lack of reliable distributional information for several key model parameters. Because many inputs were based on heterogeneous literature sources, calibrated values, or extrapolated estimates, specifying parametric distributions would have required unsupported assumptions and might have produced misleading uncertainty estimates. Uncertainty was therefore explored using deterministic one-way sensitivity analyses. Finally, the use of three age groups and the assumption of comparable effectiveness for PCV10 and PCV13 reflect modelling constraints and introduce additional uncertainty, although the WHO considers both vaccines highly immunogenic.

## 5. Conclusions

This dynamic evaluation confirms that PCV introduction in Egypt represents a high-value investment. By including all age groups, adopting a decision-relevant time horizon, incorporating outpatient disease, using realistic natural immunity assumptions, modelling herd immunity effects and serotype replacement, and applying updated economic valuation, the model estimates far larger health and economic gains than earlier under-five-only analyses. Immediate introduction maximizes benefits; delays come with substantial opportunity costs in preventable deaths and healthcare savings. Combining early PCV10 adoption with a timely transition to PCV13 can preserve strong health impacts while supporting domestic manufacturing goals and maintaining fiscal sustainability.

## Figures and Tables

**Table 4:** Pneumococcal vaccine introduction scenarios considered in the model.

Scenario	Description
1. Immediate introduction (base-case scenario)	PCV13 introduced without delay using UNICEF procurement and MICs catalytic support, with a transition to locally manufactured PCV13 beginning in year 5.
2. 5-year delay	PCV13 introduction postponed by 5 years until domestic production is established; may involve phased production through technology transfer and fill-and-finish manufacturing rather than full drug-substance production.
3. 10-year delay	PCV13 introduced after a 10-year delay, when full local production capacity is in place.
4. Immediate introduction + switch at year 5	Immediate PCV10 introduction via UNICEF procurement and MICs catalytic support, followed by a switch to locally produced PCV13 at year 5 (assuming equivalent effectiveness).
5. Immediate introduction with gradual transition	Immediate PCV10 introduction via UNICEF procurement and MICs catalytic support, with a gradual transition to local production of PCV13 between years 4 and 7; by years 8–10, all PCV13 is assumed to be procured at the local manufacturing price.

**Table 5:** Base-case results; annual budget impact in million USD.

Scenarios	Total Cases Avoided (20 Years)	Total Deaths Avoided (20 Years)	ICER/GDP Ratio (ICER)	Return on Investment	Total Annual Budget Impact Without Gavi Support	Total Annual Budget Impact with Gavi Support
Immediate introduction	38,511,202	139,451	0.16 (523.31)	23.12	124.91	120.94

**Table 6:** Cost-effectiveness outcomes over the 20-year period.

Scenarios		Total Cases Avoided	Total Deaths Avoided	ICER/GDP Ratio	ICER (Cost/DALY Averted)
1. Immediate introduction	−10% local manufacturing price	38,511,202	139,451	0.14	452.42
	+0% local manufacturing price	38,511,203	139,451	0.16	523.31
	+10% local manufacturing price	38,511,204	139,451	0.18	594.20
2. 5−year delay	−10% local manufacturing price	26,891,093	97,374	0.15	508.43
	+0% local manufacturing price	26,891,094	97,374	0.18	585.41
	+10% local manufacturing price	26,891,095	97,374	0.20	662.38
3. 10−year delay	−10% local manufacturing price	15,025,199	54,407	0.19	638.35
	+0% local manufacturing price	15,025,200	54,407	0.22	729.47
	+10% local manufacturing price	15,025,201	54,407	0.22	820.59
4. Immediate introduction + switch at year 5		38,511,202	139,451	0.12	408.30
5. Immediate introduction with gradual transition		38,511,203	139,451	0.13	419.69

**Table 7:** Budget impact and return on investment over a 20-year period (2024 USD).

Scenarios		Total Budget Impact Without Gavi Support (Million USD)	Total Budget Impact with Gavi Support (Million USD)	Return on Investment (20-Year Average)
		(Annual average)	(Annual average)	
1. Immediate introduction	−10% local manufacturing price	107.99	104.41	25.80
	+0% local manufacturing price	124.91	120.94	23.12
	+10% local manufacturing price	141.83	137.46	20.93
2. 5-year delay	−10% local manufacturing price	110.38	105.91	23.47
	+0% local manufacturing price	127.96	122.96	21.02
	+10% local manufacturing price	144.79	139.29	19.02
3. 10-year delay	−10% local manufacturing price	114.28	108.02	19.28
	+0% local manufacturing price	131.45	124.45	17.25
	+10% local manufacturing price	132.31	125.26	15.59
4. Immediate introduction + switch at year 5		103.06	102.36	27.79
5. Immediate introduction with gradual transition		105.22	103.96	27.25

**Figure A1:**
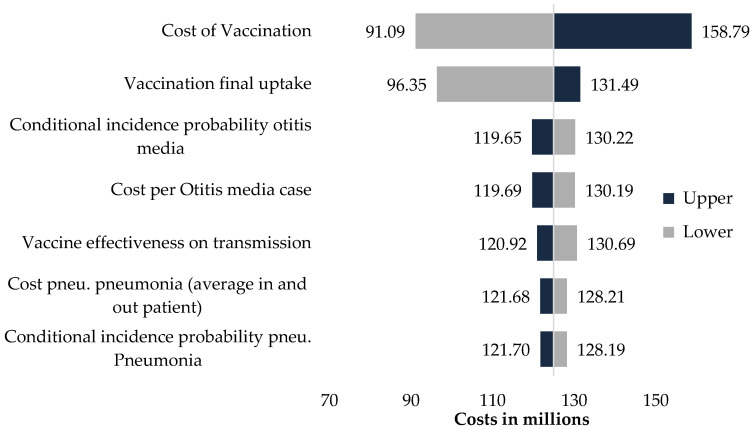
Tornado diagram.

## Data Availability

Data is available upon reasonable request to the author.

## Abbreviations

The following abbreviations are used in this manuscript:

DALYs	Disability-adjusted life years
ICER	Incremental Cost-Effectiveness Ratio
GDP	Gross Domestic Product
Gavi	Gavi the Vaccine Alliance
IPD	Invasive pneumococcal disease
LMICs	Low- and middle-income countries
LTA	Long-term arrangement
PCV10	10-valent pneumococcal conjugate vaccine
PCV13	13-valent pneumococcal conjugate vaccine
ROI	Return on Investment
S. Pn	Streptococcus Pneumoniae
WTP	Willingness to pay

## Appendix A. Dynamic Transition Model

For each age group *i*, the rate of change in the number of infectious individuals is defined by Equations (A1)–(A3). These equations allow the model to capture how vaccination alters susceptibility, transmission, and progression across the population and how indirect protection evolves over time.

(A1) $$\frac{{dI}_{i}}{dt}=\beta \cdot S_{i}\sum_{j}C_{ij}\frac{{(1-{Vac\_Eff\_Inf}_{i})\cdot Iv}_{j}+{{(1-{Nat\_Eff\_Inf}_{i})\cdot In}_{j}+I}_{j}}{N_{alive}}-\gamma I_{i}+\sum_{j}D_{ij}I_{j}$$

(A2) $$\frac{{dIv}_{i}}{dt}=\beta \cdot {Sv}_{i}\sum_{j}C_{ij}\frac{{(1-{Vac\_Eff\_Inf}_{i})\cdot Iv}_{j}+{{(1-{Nat\_Eff\_Inf}_{i})\cdot In}_{j}+I}_{j}}{N_{alive}}-\gamma {Iv}_{i}+\sum_{j}D_{ij}{Iv}_{i}$$

(A3) $$\frac{{dIn}_{i}}{dt}=\beta \cdot {Rim}_{i}\sum_{j}C_{ij}\frac{{(1-{Vac\_Eff\_Inf}_{i})\cdot Iv}_{j}+{{(1-{Nat\_Eff\_Inf}_{i})\cdot In}_{j}+I}_{j}}{N_{alive}}-\gamma {In}_{i}+\sum_{j}D_{ij}{In}_{i}$$

(A4) $$\frac{{dPD}_{i}}{dt}={PD\_inc}_{i}\cdot I_{i}\cdot \gamma -\frac{{PD}_{i}}{PD\_dur}+\sum_{j}D_{ij}{PD}_{i}$$

(A5) $$\frac{{dPDv}_{i}}{dt}=(1-{Vac\_Eff\_Disease}_{i})\cdot {PD\_inc}_{i}\cdot {Iv}_{i}\cdot \gamma -\frac{{PDv}_{i}}{PD\_dur}+\sum_{j}D_{ij}{PDv}_{i}$$

(A6) $$\frac{{dPDn}_{i}}{dt}=(1-{Nat\_Eff\_Disease}_{i})\cdot {PD\_inc}_{i}\cdot {In}_{i}\cdot \gamma -\frac{{PDn}_{i}}{PD\_dur}+\sum_{j}D_{ij}{PDn}_{i}$$

(A7) $$\frac{{d[disease]}_{i}}{dt}={frac\_[disease]}_{i}\cdot {PD}_{i}+{frac\_[disease]v}_{i}\cdot {PDv}_{i}+{frac\_[disease]n}_{i}\cdot {PDn}_{i}$$

where

- *i* and *j* index age groups.
- *β* is the transmission probability.
- *C_ij_* is the contact rate between groups *i* and *j*.
- *S_i_*, *Sv_i_*, and *Rim_i_* represent susceptible unvaccinated, susceptible vaccinated, and recovered-immune individuals.
- *I_i_*, *Iv_i_*, *In_i_* represent infectious, infectious vaccinated, and infectious recovered-immune individuals.
- *PD_i_*, *PDv_i_*, *PDn_i_* denote individuals affected with a pneumococcal disease (PD, PDv, PDn) for non-vaccinated, vaccinated, and natural immune individuals, respectively.
- *VacEffInf* and *NatEffInf* denote vaccine and natural immunity effectiveness against infection.
- *Vac_Eff_Disease_i_* denotes weighted vaccine effectiveness on the included disease.
- *Nat_Eff_Disease_i_* denotes weighted natural immunity effectiveness on the included disease.
- *PD_inc_i_* denotes the fraction of individuals that transitions to one of the included diseases.
- *PD_dur* is the average duration an individual stays ill.
- frac_[disease]_i_, frac_[disease]v_I_, frac_[disease]n_i_ denotes the fraction of the respective disease within the individuals affected with a pneumococcal disease (PD, PDv, PDn).
- *γ* is the recovery rate (1/duration of infection).
- *D* is an ageing and mortality transition matrix.
- *N_alive* is the total living population.

## Appendix B. Cost per Child Vaccinated

**Table A1:** Cost per child vaccinated using UNICEF LTA prices, 2024 USD.

Product	Cost of Vaccine			Handling		Injection Supply					Buffer Applied to Product Price (6%)	Total Vaccination Cost per Child, USD (3 Doses)
	PCV Price, USD (3 Doses)	Wastage 17% (Cost, USD)	Delivery (Cost, USD)	Vaccines 3.5%	Freight 3.2%	Injection Supply, 3 Doses (Cost, USD)	Wastage 10% (Cost, USD)	Handling AD Syringes, 16%	Freight Fees (20%)	Surcharge (8%)		
PCV13	48.00	8.16	7.02	1.68	0.22	0.16	0.016	0.03	0.03	0.0026	2.88	68.20
PCV10	10.35	1.76	7.02	0.36	0.22	0.16	0.016	0.03	0.03	0.0026	0.62	20.57

## Appendix C. Sensitivity Analyses

**Table A2:** Key results of budget-impact analysis, cost-effectiveness, and return on investment for each scenario, focusing exclusively on the health benefits for children under 5 years old.

Scenarios		Total Deaths Avoided	Total Cases Avoided	ICER/GDP Ratio	Disability Adjusted Life Years (DALYs) Averted	Total Budget Impact Without Gavi (Annual Avg)	Total Budget Impact with Gavi (Annual Avg)	Return-on Investment
	−10% local manufacturing price	46,047	20,970,490	0.27	2,160,398	133.60	130.02	7.8
Immediate	+0% local manufacturing price	46,047	20,970,490	0.30	2,160,398	150.52	146.55	6.9
	+10% local manufacturing price	46,047	20,970,490	0.33	2,160,398	167.44	163.08	6.2
	−10% local manufacturing price	32,847	14,959,141	0.29	1,419,346	99.94	96.58	7.2
5-year delay	+0% local manufacturing price	32,847	14,959,141	0.33	1,419,346	113.25	109.49	6.3
	+10% local manufacturing price	32,847	14,959,141	0.36	1,419,346	125.87	121.74	5.7
	−10% local manufacturing price	19,188	8,738,501	0.34	767,431	66.19	63.06	6.1
10-year delay	+0% local manufacturing price	19,188	8,738,501	0.38	767,431	74.84	71.34	5.3
	+10% local manufacturing price	19,188	8,738,501	0.38	767,431	75.33	71.80	4.8
Immediate, switching price from PCV10 to PCV13 in year 5, +10% local manufacturing price		46,047	20,970,490	0.25	2,160,398	130.15	129.45	
Immediate, gradually switching from PCV10 to PCV13 (years 4–7) +10% local manufacturing price		46,047	20,970,490	0.25	2,160,398	132.12	130.86	
